# Supplementary material for: Detailed Episodic Memory Depends on Concurrent Reactivation of Basic Visual Features within the Posterior Hippocampus and Early Visual Cortex
Source: Cereb Cortex Commun. 2021 Jul 16;2(3):tgab045. doi: 10.1093/texcom/tgab045 (PMC8370760; doi:10.1093/texcom/tgab045)
Supplement: ConcurReac_CereCort_SupMat_1_tgab045 [file concurreac_cerecort_supmat_1_tgab045.docx]

Supplementary Materials

Supplementary Figures

**
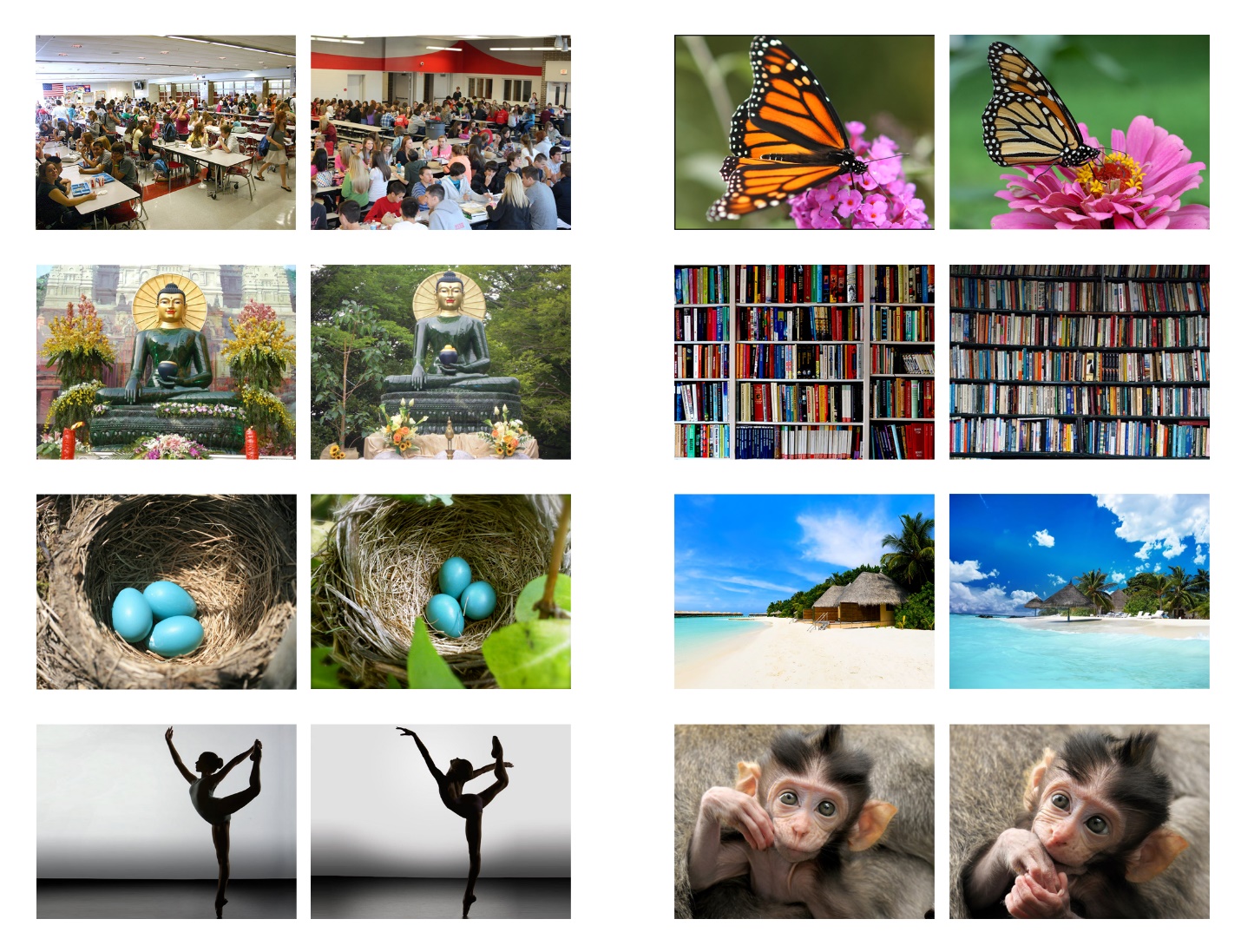
**

**Supplementary Figure 1. Example of Image Pairs.** Eight randomly selected image pairs out of the ninety total.


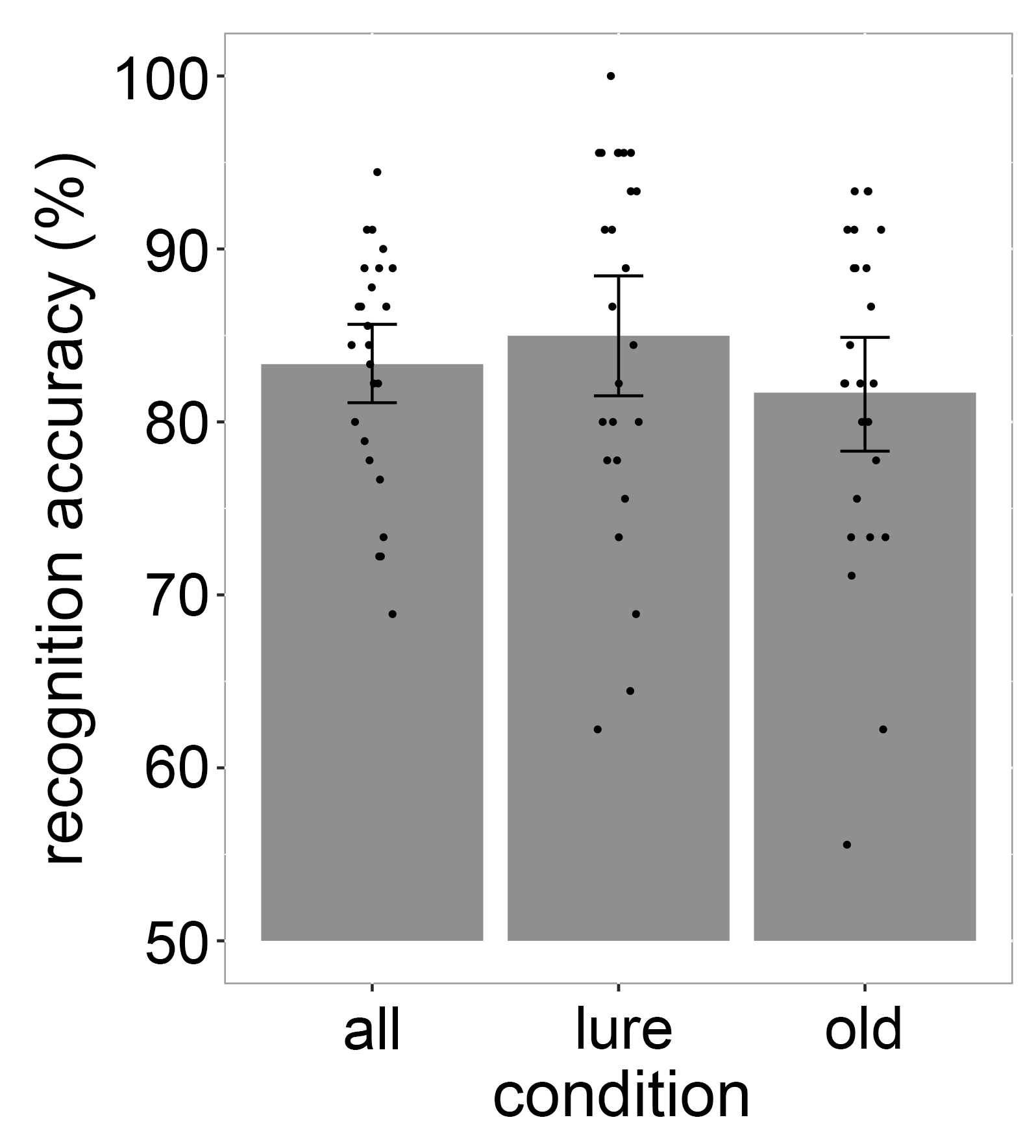


**Supplementary Figure 2. Recognition accuracy.** Each dot represents a subject. Error bars are 90% CIs.


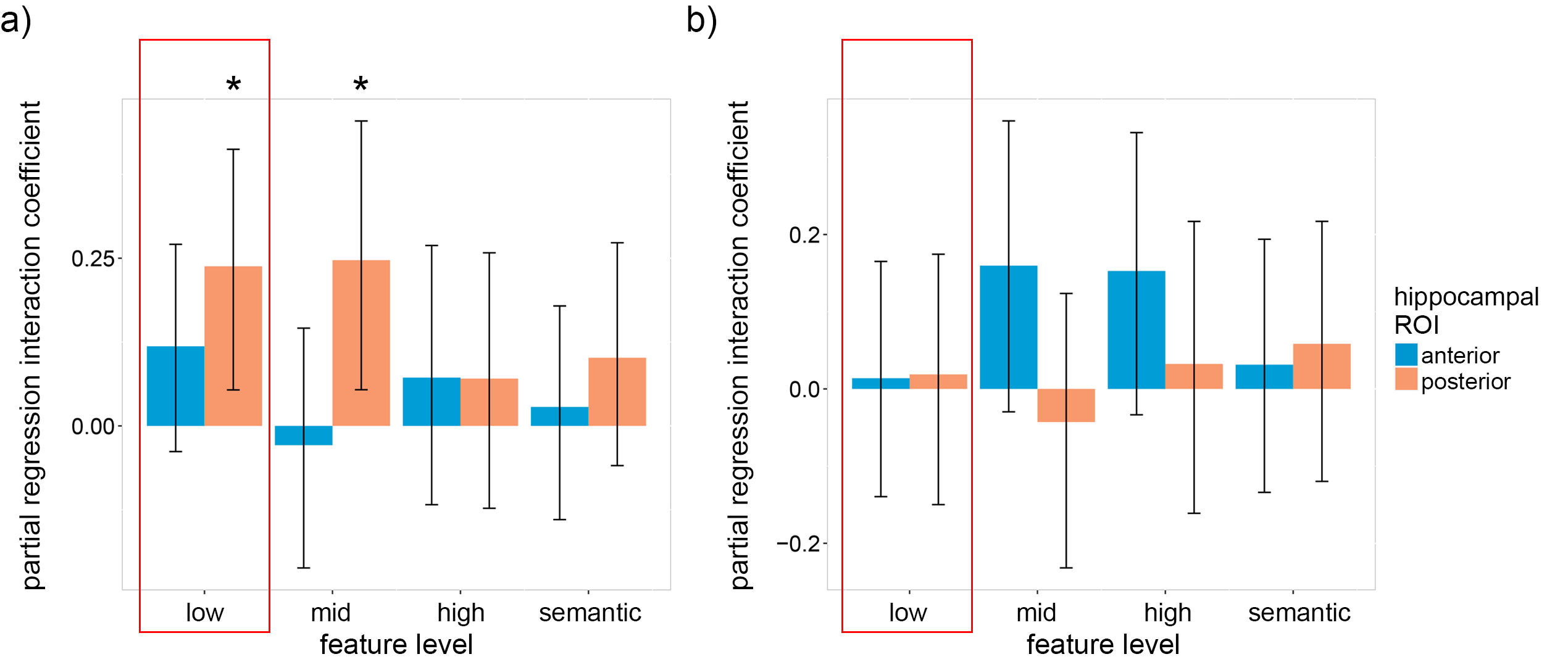


**Supplementary Figure 3. Interaction of feature-specific reactivation within the hippocampus and calcarine sulcus during recall with respect to recognition accuracy.** Within-subject partial regression coefficients for the interaction of feature-specific reactivation within the hippocampus and calcarine sulcus with respect to recognition accuracy given participant lure accuracy 1 standard deviation a) above (95%) and b) below (75%) average. The feature level along the x-axis refers to reactivation within the hippocampus. Reactivation within the calcarine sulcus was limited to low-level visual features. Error bars are 90% CIs; * indicates p < 0.05, one-tailed bootstrap; FDR corrected over visual feature levels except for low-level features because, in accordance with our hypotheses, low-level features were prioritized (indicated by the red boxes).
